# Paranoia

**DOI:** 10.1192/j.eurpsy.2022.2004

**Published:** 2022-09-01

**Authors:** M.J. Gordillo Montaño, L. Rodriguez Rodriguez, S.V. Boned Torres

**Affiliations:** Hospital Can Misses, Psychiatry, Eivissa, Spain

**Keywords:** Paranoid ideas, Epidemiology, diagnostic criteria, evolution

## Abstract

**Introduction:**

Paranoid ideas occur very often in humans (prevalence of 0.2%). According to several studies, the origin could be found in a genetic predisposition to a selective hyperdopaminergia related to the D2 receptor and dopamine neurotransmitter dysfunction.

**Objectives:**

To delve into this pathology, including origin and development, epidemiology, diagnostic criteria, clinical aspects, differential diagnosis, treatment, evolution and prognosis.

**Methods:**

We conducted a literature review of delusional disorder.

**Results:**

The disease appears in middle age, between ages 35 and 55, being slightly more frequent in women. It seems to affect more economically and educationally disadvantaged social strata, and it is more frequent in immigrants. The onset is usually progressive and insidious. Correct perception but delusional interpretation: the objectivity of what is perceived is disturbed by the subjectivity of what is registered. The delirium is usually logical, contagious, and frequently credible. Patients retain their lucidity. It is very important to make a correct differential diagnosis with schizophrenia. With regard to treatment, the therapeutic relationship with the patient will be basic. If possible, psychotherapy should be combined with pharmacological treatment (second generation antipsychotics being the treatment of choice). In general, their evolution is compatible with out-of-hospital life, being considered “odd guys”.

**Conclusions:**

The risk of suffering from Delusional Disorder during the lifetime is between 0.05 and 0.1%. This pathology constitutes 1-4% of all psychiatric admissions. Therefore, it is essential to know it in depth in order to be able to manage it properly.

**Disclosure:**

No significant relationships.

